# Somatic targeted mutation profiling of colorectal cancer precursor lesions

**DOI:** 10.1186/s12920-022-01294-w

**Published:** 2022-06-28

**Authors:** Wellington dos Santos, Mariana Bisarro dos Reis, Jun Porto, Ana Carolina de Carvalho, Marcus Matsushita, Gabriela Oliveira, Kari Syrjänen, Rui Manuel Reis, Denise Peixoto Guimarães

**Affiliations:** 1 Molecular Oncology Research Center, Barretos Cancer Hospital, Rua Antenor Duarte Vilela, 1331, Barretos, SP 14784-400 Brazil; 2 Department of Pathology, Barretos Cancer Hospital, Barretos, Brazil; 3 Research and Education Institute, Barretos Cancer Hospital, Barretos, Brazil; 4 SMW Consultants Ltd, Kaarina, Finland; 5 Department of Clinical Research, Biohit Oyj, Helsinki, Finland; 6 Life and Health Sciences Research Institute (ICVS), Medical School, University of Minho, 4710-057 Braga, Portugal; 7 ICVS/3B’s-PT Government Associate Laboratory, 4710-057 Braga, Portugal; 8 Department of Endoscopy, Barretos Cancer Hospital, Barretos, Brazil

**Keywords:** Screening, Adenoma, Serrated polyps, Mutation, Molecular profiling, Brazil

## Abstract

**Background:**

Most colorectal cancers (CRC) arise from precursor lesions. This study aimed to characterize the mutation profile of colorectal cancer precursor lesions in a Brazilian population.

**Methods:**

In total, 90 formalin-fixed paraffin-embedded colorectal precursor lesions, including 67 adenomas, 7 sessile serrated lesions, and 16 hyperplastic polyps, were analyzed by next-generation sequencing using a panel of 50 oncogenes and tumor suppressor genes. The genetic ancestry of the patients was estimated.

**Results:**

Somatic driver mutations were identified in 66.7% of cases, including alterations in *APC* (32.2%), *TP53* (20.0%), *KRAS* (18.9%), *BRAF* (13.3%) and *EGFR* (7.8%). Adenomas displayed a higher number of mutations, mainly in *APC*, compared to serrated polyps (73.1% vs. 47.8%, *p* = 0.026). Advanced adenomas had a significantly higher frequency of mutation in *KRAS* and a high overall mutation rate than early adenomas (92.9% vs. 59%, *p* = 0.006). A high degree of ancestry admixture was observed in the population studied, with a predominance of European components (mean of 73%) followed by African (mean of 11.3%). No association between genetic ancestry and type of lesions was found. The mutation profile of Brazilian colorectal precursor lesions exhibits alteration in *APC*, *KRAS*, *TP53,* and *BRAF* at different frequencies according to lesion type.

**Conclusions:**

These results bestow the knowledge of CRC's biologic history and support the potential of these biomarkers for precursor lesions detection in CRC screening of the Brazilian population.

**Supplementary Information:**

The online version contains supplementary material available at 10.1186/s12920-022-01294-w.

## Background

Colorectal cancer (CRC) is the third most incident cancer worldwide, resulting in 915,880 deaths in 2020 [1]. In Brazil, CRC ranks second in incidence in men and women [2], and a continuous increase in both incidence and mortality is expected in the coming years [3–5]. The development of colorectal cancer is a multi-stage evolution process that occurs through a progressive accumulation of molecular alterations in the colon epithelium cells, which can be transformed into pre-malignant lesions and cancer [6, 7].

Although viewed as a single disease, from the molecular and morphological point of view, CRC is a heterogeneous disease that is believed to arise mainly from two different types of precursor lesions: adenoma and serrated polyps [7, 8]. In the classic sequence of adenoma to carcinoma progression model, the development of colorectal cancer originates from aberrant crypts, progressing to early adenoma, advanced adenoma, and subsequently carcinoma [7]. Serrated polyps have recently been recognized as important precursor lesions and account for approximately 15%-30% of all cases of CRC [8–11]. According to the updated World Health Organization (WHO) classification, serrated polyps include hyperplastic polyp (HP), sessile serrated lesion (SSL), and traditional serrated adenoma (TSA) [12]. Among these lesions, the sessile serrated lesion and traditional serrated adenoma are more likely to evolve into cancer. Despite being the most frequent type of lesion, hyperplastic polyps are considered to have no malignant potential [13, 14]. Moreover, patients with proximal serrated polyps, particularly those larger than 10 mm, are associated with an increased risk of developing CRC [8].

The molecular mechanisms underlying the progression through the canonical pathway frequently comprise somatic mutations in oncogenes such as *KRAS* and tumor suppressor genes, such as *APC*, *TP53,* and *SMAD4* [7]. In addition, mutations in *BRAF* have an important play in the serrated pathway [7, 15]. Recently, our group performed a mutational portrait of Brazilian CRC patients and observed a similar molecular portrait than reported worldwide [16].

Screening for CRC can reduce incidence and mortality by detecting and removing precursor lesions [17]. This strategy is possible due to the long period of progression from a precursor lesion (adenoma) to cancer, which ranges from 7 to 10 years [17]. Colonoscopy is the most suitable and reliable diagnostic tool for CRC screening, yet it is not feasible for large-scale due to its risks and high cost. In organized population-based screening, fecal occult blood tests are preferred [18], with the FIT (fecal immunochemical test) widely used. Still, FIT is not perfect; its sensitivity for CRC ranges from 60 to 80% and only 20–30% for advanced adenoma [19]. To overcome these limitations, DNA-based analysis of body fluids–liquid biopsy–such as blood or feces, can increase the accuracy of FIT. Two commercialized assays are FDA approved for CRC screening: Epi ProColon 2.0, analyzing *SEPT9* DNA methylation in blood; Cologuard test®, stool-based that analyses *NDRG4* and *BMP3* methylation and *KRAS* mutation [20, 21].

Therefore, identifying genetic alterations in precursor lesions can lead to molecular-based strategies, improving the sensitivity, specificity, and impact of colorectal screening and surveillance programs. Nevertheless, few studies evaluated the mutation status in South America CRC precursor lesions [22, 23]. Results of the first two years (first round) of our colorectal cancer screening program (from Barretos Cancer Hospital program) in Brazil showed a successful implementation with a high test return participation rate, colonoscopy completion, and detection lesion rates. In addition, participant’s blood and FIT (fecal immunochemical test) tests has been stored in the Barretos Cancer Hospital biobank to allow future biomarker studies and consequently improve lesions detection rates [24].

Herein, we performed the mutation analysis by next-generation sequencing (NGS) of 50 oncogenes and tumor suppressor genes in colorectal cancer precursor lesions and also evaluated the genetic ancestry composition of the Brazilian samples included in the study. Somatic nucleotide variants were identified in all types of precursor lesions, most of them more prevalent in the adenoma group. Moreover, the spectrum of mutated genes was different between adenomas and serrated polyps. These results extend our knowledge of the molecular biological features of precursor lesions and the natural history of colorectal cancer.

## Methods

### Study population

This retrospective study analyzed a total of 90 formalin-fixed paraffin-embedded (FFPE) samples removed from 87 patients during diagnostic colonoscopy from 2014 to 2016 at Barretos Cancer Hospital [25]. Samples included 67 adenomas (39 early and 28 advanced adenomas), 7 sessile serrated lesions and 16 hyperplastic polyps. The 87 patients were between 49 and 88 years of age. Subjects with a personal history of familial adenomatous polyposis (FAP) or Lynch syndrome were excluded [26, 27].

The Institutional Research Board of the Barretos Cancer Hospital approved the study on Feb 4, 2016 (number ID: 1074/2016). Informed consent was waived due to the retrospective nature of this study. The study protocol conforms to the ethical guidelines of the 1975 Declaration of Helsinki.

Lesions were classified according to Paris classification [28] and histological analysis using WHO criteria [12]. Villous structures in > 25% of adenoma was required for tubulovillous adenoma. If > 75% of the adenoma has a villous architecture, it was diagnosed as villous adenoma. Adenomas were classified as advanced if > 1 cm in diameter or presented more than 25% of villous structures in histology or high-grade intraepithelial neoplasia. Table 1 summarizes the histopathological data of the 90 samples.

**Table 1:** Clinical, morphological and histopathological features of the lesions analyzed in the study

Characteristic	Number of cases	(%)
Age (mean ± sd)	62.9 ± 9.09	–
Gender		
Male	43	49.4
Female	44	50.6
Histology		
Adenoma	67	74.4
Tubular	51	56.7
Tubulovillous	13	14.4
Villous	3	3.3
Serrated polyps	23	25.6
Hyperplastic polyps	16	17.8
MVHP	9	10.0
GCHP	7	7.8
Sessile Serrated Lesion	7	7.8
Morphology		
Polypoid	76	84.4
Non polypoid	14	15.6
Location		
Proximal colon	40	44.4
Distal colon	50	55.6
Size mm		
< 10	69	76.7
≥ 10	21	23.3

*Sd* standard deviation

N = 87 patients and 90 precursor lesions

### DNA isolation

DNA was isolated from FFPE tissue as previously reported [23]. Hematoxylin and eosin stained slides were reviewed by a pathologist and the contents of precursor lesions samples were more than 50%. The lesion area was delimited and macrodissected from six 10-μm-thick unstained tissue sections. Xylene and ethanol (100%, 70%, 50%) were used for paraffin removal.

FFPE genomic DNA was extracted using the QiaAmp DNA micro Kit (Qiagen, Hilden, Germany) according to the manufacturer’s instructions. DNA concentration was determined using Qubit™ dsDNA HS assay kit (Thermo Fisher Scientific, Eugene, Oregon, USA) on the Qubit 2.0 Fluorometer (Thermo Fisher Scientific).

### Library preparation and sequencing

Next-generation sequencing library preparation was conducted to amplify 10 ng of genomic DNA using AmpliSeq™ Cancer HotSpot Panel v2 panel kit (Thermo Fisher Scientific) and was performed with Ion Ampliseq™ Kit for Chef DL8 in the Ion Chef instrument. This panel includes primer for regions of the following 50 cancer driver genes—*SMARCB1*, *RB1*, *TP53*, *ERBB4*, *FBXW7*, *BRAF*, *KIT*, *GNAS*, *HRAS*, *EGFR*, *PDGFRA*, *PIK3CA*, *CDKN2A*, *ERBB2*, *ABL1*, *JAK2*, *KRAS*, *NRAS*, *NOTCH1*, *ATM*, *FGFR1*, *STK11*, *PTPN11*, *APC*, *SMAD4*, *PTEN*, *SMO*, *CTNNB1*, *RET*, *IDH2*, *SRC*, *EZH2*, *VHL*, *MPL*, *NPM1*, *FLT3*, *FGFR3*, *CDH1*, *KDR*, *HNF1A*, *MLH1*, *ALK*, *IDH1*, *GNAQ*, *AKT1*, *JAK3*, *FGFR2*, *GNA11*, *MET*, *CSF1R*. Pooled libraries were submitted to emulsion PCR, enrichment of beads containing the template and chip loading in the Ion Chef instrument using Ion PGM™ Hi-Q™ View Chef Kit according to the manufacturer’s instructions (Thermo Fisher Scientific, Waltham, MA). The final library was sequenced in an Ion 318 v2 chip on Ion Torrent PGM using Ion PGM™ Hi-Q™ View supplies.

### Data analysis

Sequencing data were processed in the Ion PGM™ Torrent Server and generated reads were aligned to the reference genome (hg19) using TMAP (Torrent Mapping Alignment Program) in the Torrent Suite™ Software (ThermoFisher). Variant calling and annotation were performed using the Ion Reporter™ Software (version 5.10).

Only variants with sequencing depth of at least 200 × and variant allele frequency (VAF) > 10% were retained. Intronic and synonymous variants were filtered out, as well variants with frequency higher than 1% in population database (ExAC), available in The Cancer Genome Interpreter tool (CGI). This platform was also employed to verify the status of driver variants and only known variants or predicted as driver in colorectal cancer-related genes were retained. In addition, the remaining variants with frequency higher than 1% in the Brazilian genomic variants database (ABraOM) were excluded from further analysis.

### Validation

Samples harboring the *BRAF* V600E mutation (n = 3) were selected for the qualitative validation of the variant with the real-time PCR assay cobas 4800 *BRAF* V600 Mutation Test (Roche Molecular Diagnostics). The test was performed using cobas 4800 System according to the manufacturer’s instructions.

### Genetic ancestry analysis

DNA isolation from peripheral blood samples was performed using the QIAmp DNA Blood Mini Kit (QIAGEN, Hilden, Germany) following the manufacturer’s instructions and by Biobank Barretos procedures [29]. DNA concentration was determined using NanoDrop™ Spectrophotometer (Thermo Scientific).

The genetic ancestry of 81 patients with colorectal precursor lesions with available blood was determined by 46 autosomal ancestry informative markers (AIMs), which consist of insertion-deletion polymorphisms (INDELs) as described [30]. A multiplex PCR was followed by a fragment analysis performed by ABI 3500xL Genetic Analyzer (Applied Biosystems). The analysis of genotypes was performed with GeneMapper Software v4.1(Applied Biosystems).

Genetic data of the Human Genome Diversity Project Center d’Etude du Polymorphisme Humain (HGDP-CEPH) [31] was used for the classification of the proportion of ancestry for each of the main populations: African, European, East Asian and, Native American using the Structure Software v2.3.4 [32, 33].

### Statistical analysis

Descriptive statistics were expressed in number, percentage, mean and standard deviation. The Chi-square test and Fisher's exact test were used to compare the mutation rates between the different pathological and clinical features of colorectal lesions. The association of the genetic ancestry component (AFR-African, EUR-European, EAS-Asian, NAM-Native American) with precursor lesions was performed using the Kruskal–Wallis test. *P* values were adjusted by Bonferroni correction for multiple comparisons analysis.

All statistical analyses were performed using SPSS software (v.21) and R software (v. 3.6.1).

## Results

### Summary of identified driver variants

The mean amplicon sequence coverage of 200 × used in AmpliSeq Cancer HotSpot v2 panel was 99.27%. The average coverage per amplicon, number of mapped reads, on target percent and mean depth per sample are shown in Additional file 1: Fig. S1 and Additional file 4: Table S1.

Among the 90 lesions included in this study, 60 (66.7%) showed at least one driver variant. Overall, a total of 124 somatic non-synonymous driver variants were identified in 16 genes. Sequencing of colorectal lesions showed a mean of 1.4 (range of 1–8) driver mutations per lesion. The following mutation frequencies were identified: 33.3% (30/90) of lesions showed no driver mutations, 34.4% (31/90) of lesions carried one mutation per case, 14.4% (13/90) had two, 5.6% (5/90) had three mutations and 11 samples (12.2%) had four or more mutations.

**Fig. 1:**
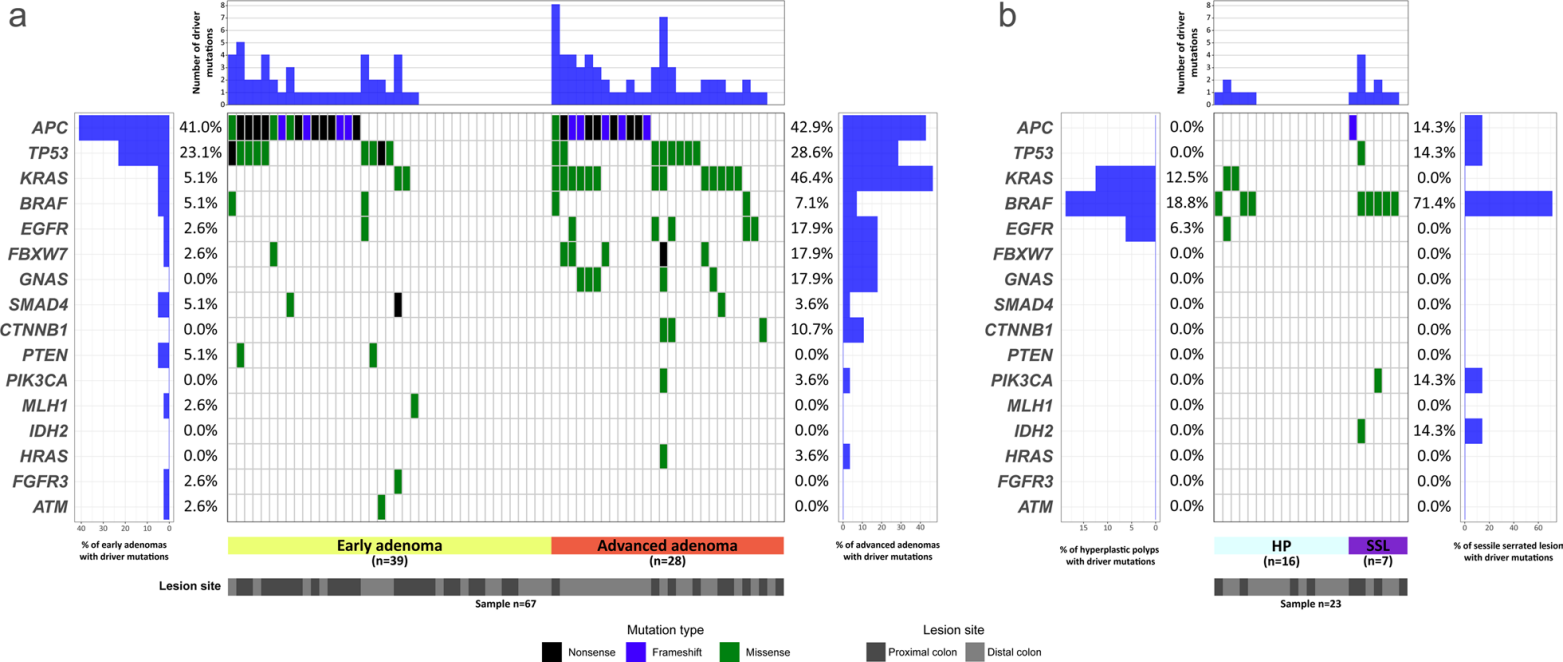
Waterfall plot of the driver mutation spectrum of colorectal cancer precursor lesions. Plots show the frequency of samples mutated for adenoma lesions (**a**) and serrated polyps (**b**). The upper panel demonstrates the frequency of mutation for each sample. Left panel shows the frequency of samples harboring mutations according to the gene. The lower panel indicates the lesion site and classification of the lesion. Adenomas are more likely to harbor mutations in *APC* while serrated polyps frequently harbor *BRAF* mutations. HP: hyperplastic polyp; SSL: sessile serrated lesion

The spectrum of identified mutations included 92 missense, 20 nonsense, 10 frameshift and 2 splice site variants. *APC* alterations were the most common in our samples (32.2%), followed by *TP53* (20.0%), *KRAS* (18.9%), *BRAF* (13.3%) and *EGFR* (7.8%) (Fig. 1, Additional file 2: Fig. S2 and Additional file 5: Table S2 and Additional file 6: Table S3). Mutations in three selected *BRAF* V600E mutated cases were validated using cobas® 4800 *BRAF* V600 Mutation Test.

### Variants in colorectal lesions exhibit differences based on lesion histological type

We further analyzed the variants according to the lesion histological type and localization (Fig. 1). The frequency of driver mutations in adenomas was slightly higher (73.1% of cases with at least one variant) when compared to serrated polyps (47.8%, *p* = 0.117) (Table 2). In addition, a mean of 1.6 driver mutations per case were identified in adenomas and 0.7 in serrated polyps (*p* = 0.01).

**Table 2:** Comparison of driver mutations frequency among different features of colorectal lesions

	Driver mutations				Adjusted *p* value
	No		Yes		
	n	%	n	%	
Histological type					
Adenomas	18	26.9	49	73.1	0.117^a^
Serrated polyps	12	52.2	11	47.8	
Adenoma					
Early adenoma	16	42.0	23	59.0	0.006^a^
Advanced adenoma	2	7.1	26	92.9	
Serrated polyps					
Hyperplastic polyps	11	68.7	5	31.3	0.081^b^
Sessile serrated lesions	1	14.3	6	85.7	

^a^χ^2^ Test

^b^Fisher's exact test; *p* values were adjusted for multiple comparisons with Bonferroni method

Number of variants were significantly higher in advanced adenomas than early adenomas with 2.2 *vs*.1.2 variants per lesion (*p* = 0.03), respectively, and were detected in 92.9% and 59.0% of the lesions, respectively (*p* = 0.006). Slightly differences were also observed between serrated polyps, although not significant, with more variants detected in sessile serrated lesions when compared to hyperplastic polyps (85.7% *vs* 31.3%, *p* = 0.081). The mean numbers of mutations were 1.4 in SSL and 0.4 in hyperplastic polyps (*p* = 0.096) (Additional file 3: Fig. S3).

Differences in frequency of variants in the 16 genes between adenoma, sessile serrated lesions and hyperplastic polyps are shown in Table 3. The differences observed were higher frequency of *APC* mutations in adenomas when compared to serrated sessile lesion and hyperplastic polyp, while variants in *BRAF* were more prevalent in sessile serrated lesions (Table 3). When comparing early and advanced adenomas, a higher frequency of mutations in *KRAS* was observed in the late stage of the lesion (*p* = 0.001, Fig. 1a, Additional file 7: Table S4). Although we observed a higher frequency in *BRAF* when comparing hyperplastic polyps and SSL (Fig. 1b, Additional file 7: Table S4), this difference was not significant when adjusted analysis were performed.

**Table 3:** Frequency of the most common altered genes in different groups of lesions

Gene	Adenoma		HP		SSL		Adjusted *p* value
	n	(%)	n	(%)	n	(%)	
*APC*	28	41.8	0	0.0	1	14.3	0.016^a^
*TP53*	17	25.4	0	0.0	1	14.3	0.816^a^
*KRAS*	15	22.4	2	12.5	0	0.0	> 0.999^a^
*BRAF*	4	6.0	3	18.8	5	71.4	0.001^a^
*EGFR*	6	9.0	1	6.3	0	0.0	> 0.999^a^
*FBXW7*	6	9.0	0	0.0	0	0.0	0.752^a^
*GNAS*	5	7.5	0	0.0	0	0.0	0.721^a^
*SMAD4*	3	4.5	0	0.0	0	0.0	> 0.999^a^
*CTNNB1*	3	4.5	0	0.0	0	0.0	> 0.999^a^
*PTEN*	2	3.0	0	0.0	0	0.0	> 0.999^a^
*PIK3CA*	1	1.5	0	0.0	1	14.3	> 0.999^a^
*MLH1*	1	1.5	0	0.0	0	0.0	> 0.999^a^
*IDH2*	0	0.0	0	0.0	1	14.3	> 0.999^a^
*HRAS*	1	1.5	0	0.0	0	0.0	> 0.999^a^
*FGFR3*	1	1.5	0	0.0	0	0.0	> 0.999^a^
*ATM*	1	1.5	0	0.0	0	0.0	> 0.999^a^

^a^Fisher's exact test; *p values* were adjusted for multiple comparisons with Bonferroni method

Analysis comparing mutational status in adenomas according to the location of lesions revealed that *GNAS* (*p* = 0.003) was significantly mutated in rectal adenomas (Additional file 8: Table S5). In serrated polyps, none of the genes were significant altered according to the location (Additional file 9: Table S6).

### Molecular pathways associated to colorectal cancer in precursor lesions

**Fig. 2:**
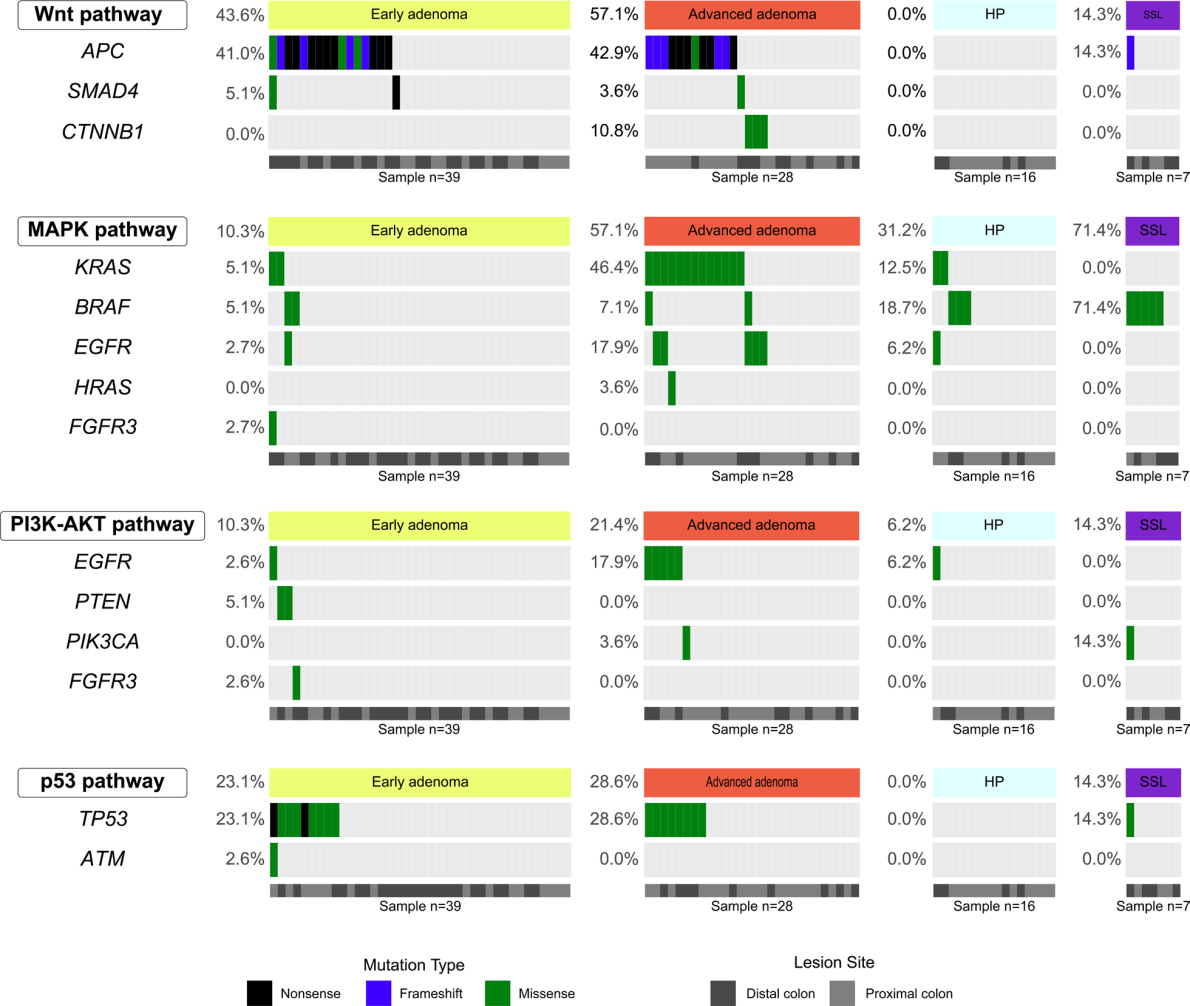
Colorectal cancer associated pathways in precursor lesions. Genetic alterations in precursor lesions occur in genes of the Wnt, MAPK, PI3K-AKT and p53 pathways

Several genes associated with important signaling pathways in cancer were identified as frequently mutated (Fig. 2). The Wnt pathway (*APC*, *SMAD4* and, *CTNNB1* genes) was the signaling pathway with the highest frequency of alterations (37.8%). The highest frequency of alteration in this pathway was found among samples of adenoma group when compared to serrated polyps (49.3% vs 4.3%, *p* < 0.001), with increased frequency of mutations in advanced adenomas, followed by early adenomas, SSLs and hyperplastic polyps (*p* = 0.001, Fig. 2).

*KRAS*, *BRAF*, *EGFR*, *HRAS* and *FGFR3* genes of MAPK pathway were mutated in 33.3% of the cases. *BRAF* and *KRAS* genes were mutually exclusive mutated, except for one advanced adenoma. Mutations were more frequently identified among SSLs, followed by advanced adenomas, hyperplastic polyps, and early adenomas (*p* < 0.001).

Regarding the PI3K-AKT pathway, mutations in the *EGFR*, *PTEN*, *PIK3CA,* and *FGFR3* genes were found in 13.3% of the cases. Mutations in genes involved in the p53 pathway (*TP53* and *ATM*) were found in 20% of the lesions, mostly with mutations in the *TP53* gene.

### Genetic ancestry of patients with colorectal lesions

**Fig. 3:**
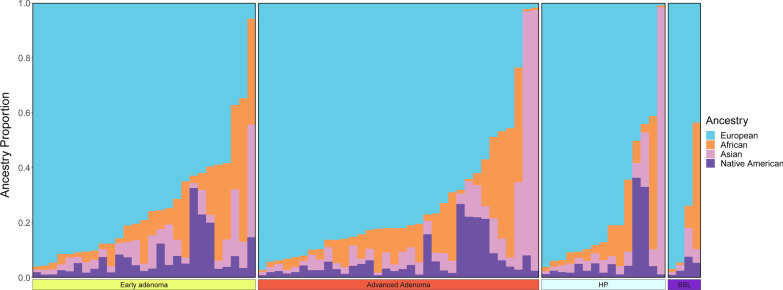
Individual ancestry estimates for the precursor lesions. Individuals are represented by a vertical bar and colors indicate the proportion of ancestry

The genetic ancestry component was obtained for 80 patients (91.9%) included in the study. The results indicated a high admixture and heterogeneity of the ancestry proportion of Brazilian samples, with the mean of ancestral proportions as follow: 73% (SD = 23.6%) for EUR, 11.3% (SD = 13.2%) for AFR, 9.1% (SD = 18.0%) for EAS and 6.6% (SD = 7.9%) for NAM (Fig. 3). We further compared the average genetic ancestry according to the lesion type, but no significant association was found (Additional file 10: Table S7). According to the mutation status, no differences were observed between the patient’s ancestry (Additional file 11: Table S8).

## Discussion

In the present study, we carried out a molecular profile of 50 cancer-related genes in precursor lesions of CRC. Adenomas exhibited mutations in genes already known to be involved in colorectal carcinogenesis, such as *APC*, *KRAS*, *TP53,* and *FBXW7*. On the other hand, serrated polyps showed low frequency in *APC* and *TP53* genes and a high frequency of *BRAF* gene mutations.

Our findings corroborate the molecular differences previously reported in these two major distinct pathways of carcinogenesis [7]. According to the classic adenoma-carcinoma progression model, the progressive accumulation of genetic alterations leads to carcinoma development from the normal mucosa [34]. Recent studies have added complexity to this model, demonstrating the presence of molecular heterogeneity in the early stages of the development of colorectal lesions and mutations in several genes considered drivers for CRC [35–38]. As expected, we found a higher average of driver mutations in advanced adenomas than in early adenomas. The acquisition rate of mutations is increased in adenomas than normal tissue, and the mutational burden in advanced adenomas has been reported to be similar to cancer tissues, even when only driver mutations are analyzed [35, 36, 39].

We also reported a slightly lower frequency of mutations among serrated polyps when compared to adenomas. Few studies addressed this issue [38, 40]. When comparing only SSL and adenomas, these authors show no difference in the frequency of mutations among these groups, likewise our study. Further, we also observed a slightly higher mutation frequency in SSLs than in hyperplastic polyps, which are lesions with lower malignancy potential. Recently, hyperplastic polyps and serrated sessile lesions were associated with the Consensus Molecular Subtype 1 (CMS1), which often has microsatellite instability (MSI) and hypermutation [41, 42].

Additionally, we found a significant difference in the Wnt, MAPK, PI3K-AKT, and p53 signaling pathways between adenomas and serrated polyps. Alterations in the Wnt pathway are an initial event in the adenoma-carcinoma progression, predominantly due to mutations in the *APC* (40.3% to 80.0%) followed by the *CTNNB1* gene (11.9–20.0%) [43–45]. Our study found a lower frequency of *APC* (41.8%) and *CTNNB1* (3.3%) mutations, which can be because we did not analyze the whole coding sequence, but the major hotspot regions of both genes. In the serrated polyps pathway, the Wnt signaling is reported to be less targeted [40, 46], following our findings.

Activation of the MAPK pathway is also observed in CRC, with mutations mainly found in *KRAS* and *BRAF* oncogenes [47, 48]. We found 33.3% of our samples harboring mutations in this pathway, with mutations in the *KRAS* gene slightly more frequent in the adenoma group (22.4%) and *BRAF* predominantly present in the SSL group. In the adenoma group, the *KRAS* mutation frequency is within the variation observed in other studies (10.7% to 60.0%) [38, 43, 49, 50]. For the Brazilian population, previous reports on the frequency of *KRAS* mutation in adenomas have reported a lower frequency than we found (13.6%) [23]. This difference could be explained by the higher sensitivity of NGS used in this study compared to Sanger sequencing to detect low-frequency variants [51, 52]. We also observed a higher frequency of mutations in the *KRAS* gene in advanced adenomas than in early adenomas, similar to previous studies [38, 53], including reports on the Brazilian population [23].

A high frequency of mutations in the MAPK pathway genes was observed in serrated polyps, mainly due to the activating *BRAF* gene mutations in SSLs. This is consistent with a previously reported frequency of *BRAF* mutations in our population [23]. Mutations in the *BRAF* gene have been consistently related to SSL with a high frequency of samples (8.7–88%) harboring mutations [23, 40, 45, 50, 54]. Interestingly, the main activating mutation *BRAF* V600E was found only in serrated polyps, as previously reported [23, 49].

Mutations in *TP53* are generally observed during the transition from adenoma to carcinoma [35, 55, 56]. Recent studies reported a lower frequency of *TP53* mutations in early or low growth rate adenomas and a higher mutation frequency during the progression of early to advanced adenomas [38, 49]. No significant difference was observed between early and advanced adenomas in our data. Nevertheless, our results agree with the Vogelstein model, where *TP53* is associated with the adenoma-carcinoma transition. The frequency of mutations in adenomas was lower when compared to the frequency of mutations in CRC cases previously reported in our population (25.4% in adenomas vs. 56.0% in cancer) [16]. Besides, mutations in *TP53* in the serrated polyps were found only in SSLs, which was already described [40, 57].

According to previous reports, genes of the PI3K-AKT pathway were also mutated in our samples [47, 58]. This pathway may present mutations in precursor lesions, focusing on advanced adenomas or traditional sessile adenomas [59], suggesting a role in the late steps of both adenoma-carcinoma and serrated pathways progression. In agreement with these data, we observed a slightly higher frequency of mutations in genes of this pathway in advanced adenomas than in early adenomas. In the advanced adenomas, we found 3.6% of samples harboring mutations in *PIK3CA*. Mutations in this gene are found in regions of carcinoma in situ [36]. However, it is not an initial event during clonal diversification in carcinogenesis, as observed in studies of clonal evolution in CRC [35, 56, 60]. Also, *PIK3CA* mutations are found in cancer-associated adenomas (20.0–30.0%) [36, 43], or lower frequency in advanced adenomas (3.2%) [61], similar to the frequency observed in our study.

The presence of mutations in the *GNAS* gene is frequent in CRC [7] and has been reported in advanced adenomas [35, 54, 62]. Although mutations in *GNAS* in serrated polyps have already been reported, its frequency is not high and is related to more advanced lesions [63]. Corroborating these data, we identified mutations in this gene only in advanced adenomas samples and absent in serrated polyps.

Previous studies have reported that polyps and colorectal cancer are more frequent among African Americans than non-Hispanic Whites [64–66]. In the present study, as expected [30, 67, 68], we observed a high heterogeneity of the ancestry proportions in our study population, yet, we did not find any difference between genetic ancestry and the groups of precursor lesions evaluated. This result could be due to the small number of cases within each group analyzed. Nevertheless, this is the first study to analyze the mutation profile of CRC precursor lesions in this high admixture population, contributing to overcoming disparities and reducing inequalities in the knowledge of colorectal genomic studies [69, 70]. Yet, the identification of somatic alteration in a heterogeneous ancestry population may have distinct medical significance across population groups [69]. Despite major findings, our study’s limitations lie in the absence of paired normal tissue samples compared to the profile mutation of lesion samples. To overcome this issue, the variants identified were filtered in databases, such as ABraOM (Brazilian population) and ExAC (international population). Also, the nature of the targeted sequencing, which does not cover the whole coding sequencing of the cancer genes, could underestimate the mutation frequencies. Finally, the absence of critical CRC-related genes, such as *TCF7L2* and *FAM123B* [58], could limit our results’ interpretation.

## Conclusions

In summary, our study reports the mutation profile of colorectal precursor lesions in Brazilian patients for the first time. We observed the highest mutation frequency in known CRC driver genes, including *APC*, *TP53,* *KRAS,* and *BRAF*, with differences according to the type of lesion analyzed, with a higher rate of mutations in adenomas. Moreover, a higher number of mutations were found in advanced adenomas compared to early adenomas and in SSL compared to hyperplastic polyps. Collectively, these findings support the potential of these biomarkers for precursor lesions detection in CRC screening of the Brazilian population.

## Supplementary Information

**Additional file 1: Figure S1.** Boxplots reporting the median coverage of the 207 amplicons sequenced across the 50 genes for all precursor lesion samples. The average depth of all amplicons was 1631.5x per sample (ranging from 0x to 13823x).

**Additional file 2: Figure S2.** Lollipop plots showing the distribution of mutations in 16 genes identified altered in colorectal cancer precursor lesions. The Y-axis represents the number of mutations at each residue. Truncating mutations are represented by black circles and green circles indicated a missense mutation. Plot from cBioPortal (http://www.cBioPortal).

**Additional file 3: Figure S3.** Number of driver mutations detected in the sequencing differs between the four classes of precursor lesions (Kruskal-Wallis test p < 0.001). The boxplot shows the median number of mutations observed across the early adenomas, advanced adenomas, hyperplastic polyps and sessile serrated lesion. Mann-Whitney test was used to determine the statistical significance with Bonferroni multiple comparisons correction: *p < 0.05; **p < 0.001.

**Additional file 4: Table S1.** Sequencing metrics per sample.

**Additional file 5: Table S2.** Frequency of detected mutations in colorectal cancer precursor lesions.

**Additional file 6: Table S3.** Detected mutations in colorectal cancer precursor lesions per patient.

**Additional file 7: Table S4.** Frequency of the most common altered genes in early and advanced adenoma, and serrated polyps.

**Additional file 8: Table S5.** Association between mutation status and location for adenoma samples.

**Additional file 9: Table S6.** Association between mutation status and location for serrated polyp samples.

**Additional file 10: Table S7.** Ancestry background proportions for all four ethnic groups according to lesion type.

**Additional file 11: Table S8.** Ancestry background proportions for all four ethnic groups according to mutation status.

## Data Availability

The original contributions presented in the study are publicly available. This data can be found here: http://www.ncbi.nlm.nih.gov/bioproject/772782 (BIOPROJECT accession number: PRJNA772782).

## Abbreviations

ABraOM: Brazilian genomic variants database
AFR: African
AIMs: Autosomal ancestry informative markers
CGI: The Cancer Genome Interpreter
CMS1: Consensus Molecular Subtype 1
CRC: Colorectal cancer
EAS: Asian
EUR: European
ExAC: Exome Aggregation Consortium
FAP: Familial adenomatous polyposis
FIT: Fecal Immunochemical Test
FFPE: Formalin-fixed paraffin-embedded
HGDP-CEPH: Human Genome Diversity Project Center d’Etude du Polymorphisme Humain
HP: Hyperplastic polyp
INDELs: Insertion-deletion polymorphisms
MSI: Microsatellite instability
NAM: Native American
NGS: Next-generation sequencing
PCR: Polymerase Chain Reaction
SD: Standard Deviation
SSL: Sessile serrated lesion
TSA: Traditional serrated adenoma
TMAP: Torrent Mapping Alignment Program
VAF: Variant allele frequency
WHO: World Health Organization
