# Supplementary material for: Somatic targeted mutation profiling of colorectal cancer precursor lesions
Source: BMC Med Genomics. 2022 Jun 28;15:143. doi: 10.1186/s12920-022-01294-w (PMC9238170; doi:10.1186/s12920-022-01294-w)
Supplement: Supplementary file 1 — Additional file 1: Figure S1. Boxplots reporting the median coverage of the 207 amplicons sequenced across the 50 genes for all precursor lesion samples. The average depth of all amplicons was 1631.5x per sample (ranging from 0x to 13823x). [file 12920_2022_1294_MOESM1_ESM.pdf]

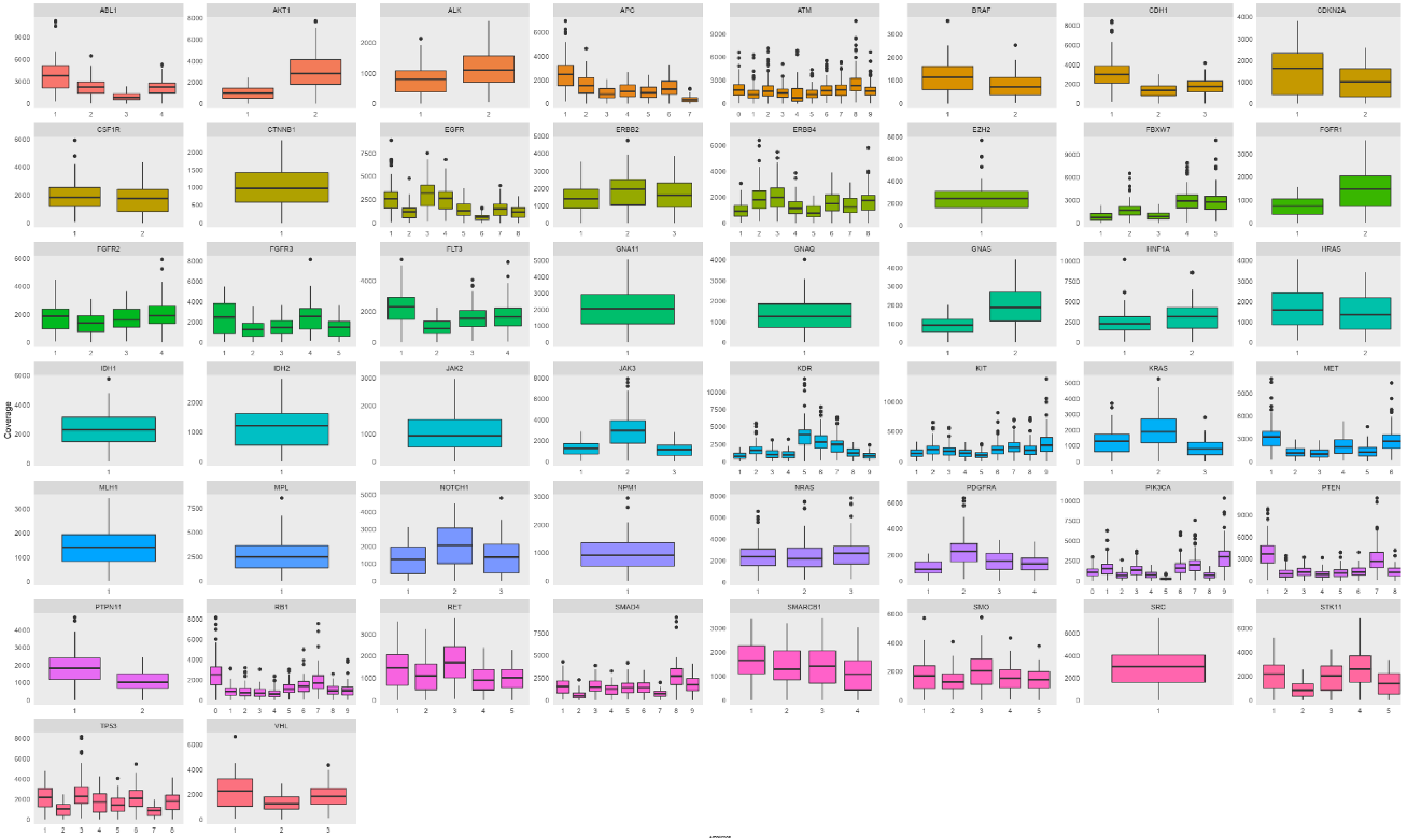

Figure S1. Boxplots reporting the median coverage of the 207 amplicons sequenced across the 50 genes for all precursor lesion samples. The average depth of all amplicons was 1631.5x per sample (ranging from 0x to 13823x).
