# Supplementary figures and images for: Somatic targeted mutation profiling of colorectal cancer precursor lesions
Source: BMC Med Genomics. 2022 Jun 28;15:143. doi: 10.1186/s12920-022-01294-w (PMC9238170; doi:10.1186/s12920-022-01294-w)

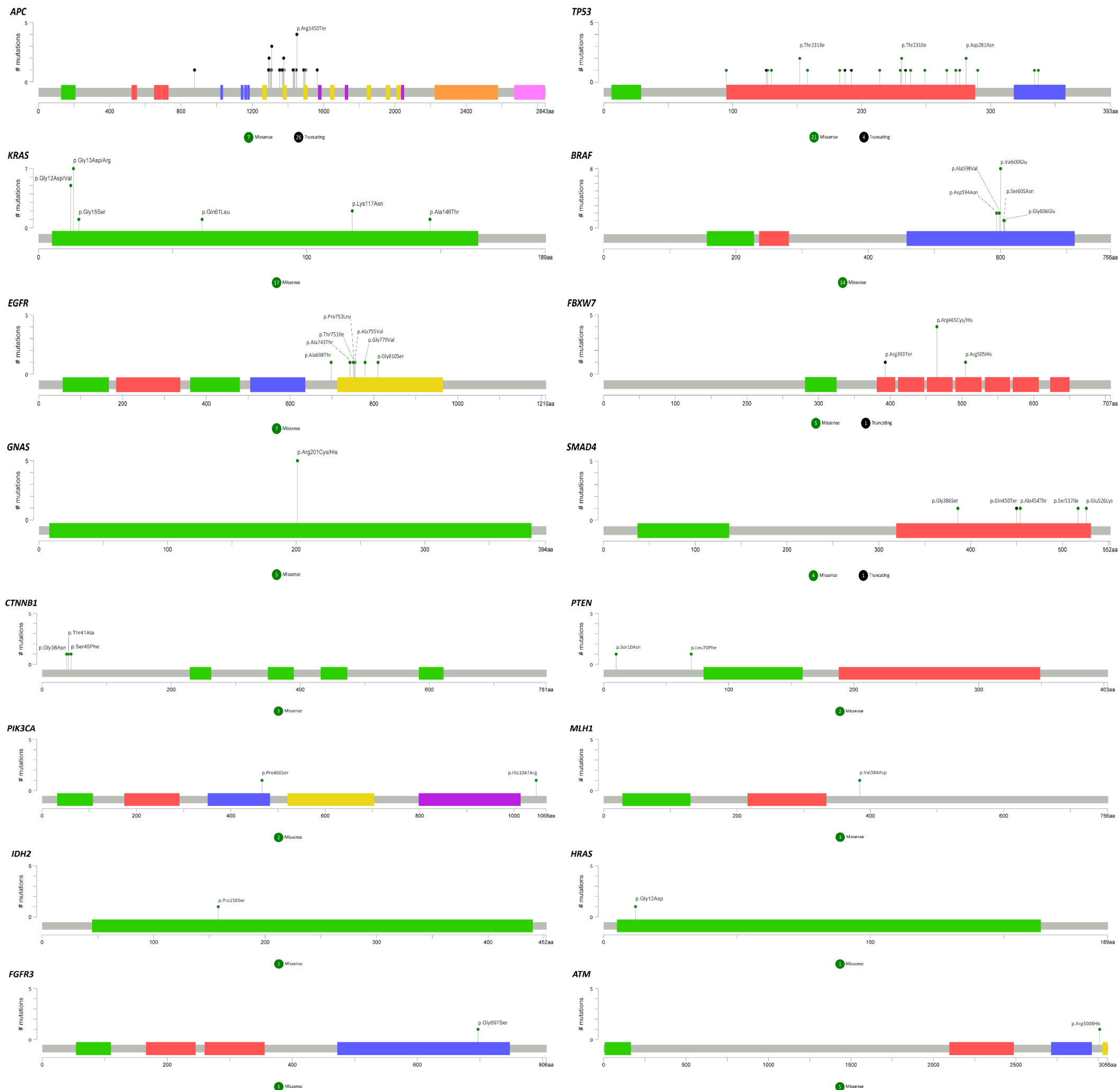

Supplement: Supplementary file 2 — Additional file 2: Figure S2. Lollipop plots showing the distribution of mutations in 16 genes identified altered in colorectal cancer precursor lesions. The Y-axis represents the number of mutations at each residue. Truncating mutations are represented by black circles and green circles indicated a missense mutation. Plot from cBioPortal (http://www.cBioPortal). [file 12920_2022_1294_MOESM2_ESM.pdf]
