# Supplementary material for: Somatic targeted mutation profiling of colorectal cancer precursor lesions
Source: BMC Med Genomics. 2022 Jun 28;15:143. doi: 10.1186/s12920-022-01294-w (PMC9238170; doi:10.1186/s12920-022-01294-w)
Supplement: Supplementary file 3 — Additional file 3: Figure S3. Number of driver mutations detected in the sequencing differs between the four classes of precursor lesions (Kruskal-Wallis test p < 0.001). The boxplot shows the median number of mutations observed across the early adenomas, advanced adenomas, hyperplastic polyps and sessile serrated lesion. Mann-Whitney test was used to determine the statistical significance with Bonferroni multiple comparisons correction: *p < 0.05; **p < 0.001. [file 12920_2022_1294_MOESM3_ESM.pdf]

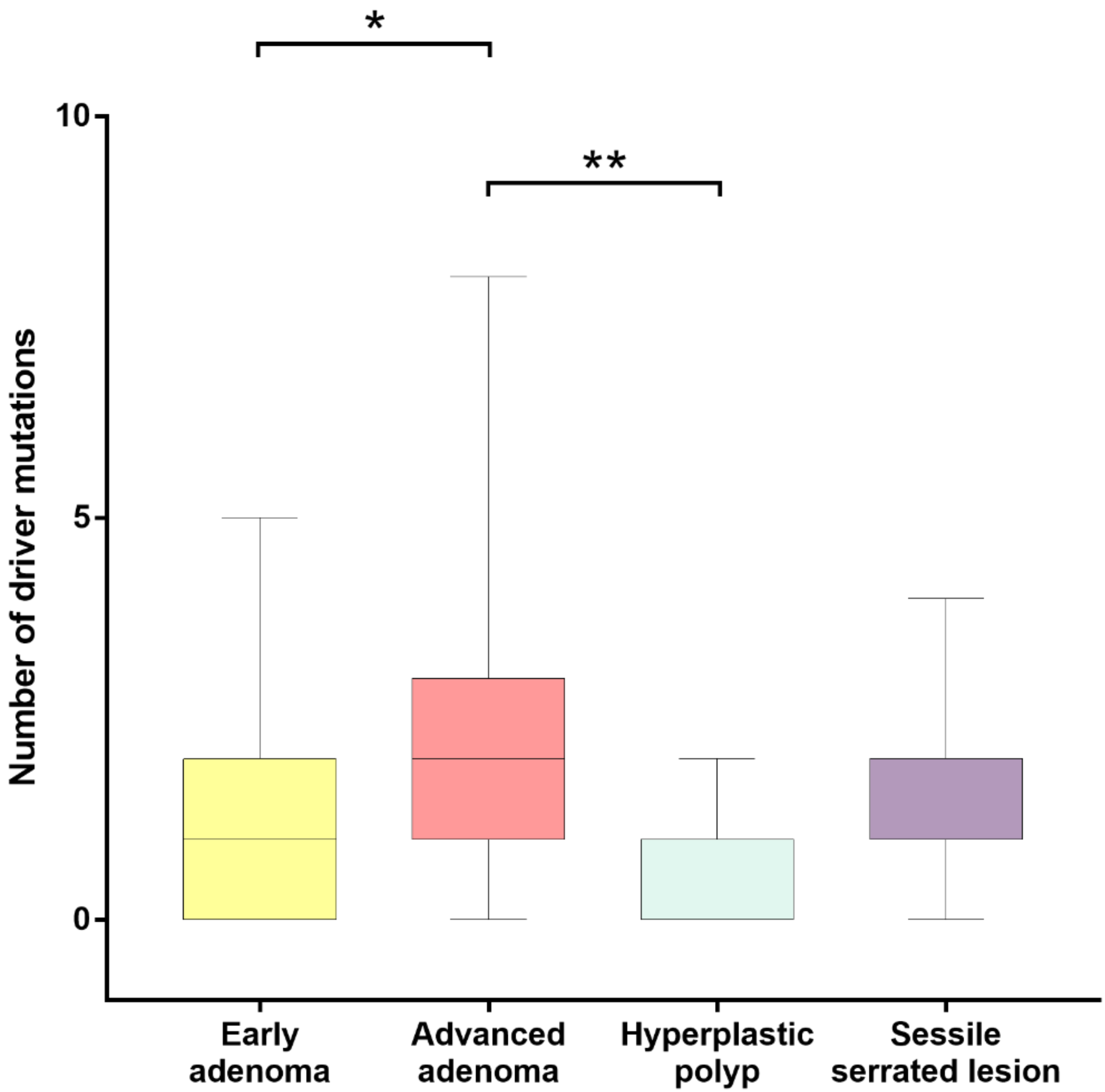

Figure S3. Number of driver mutations detected in the sequencing differs between the four classes of precursor lesions (Kruskal-Wallis test p<0,001). The boxplot shows the median number of mutations observed across the early adenomas, advanced adenomas, hyperplastic polyps and sessile serrated lesion. Mann-Whitney test was used to determine the statistical significance with Bonferroni multiple comparisons correction: *p<0.05; **p<0.001.
